# Association between triglyceride glucose index and carotid artery plaque in different glucose metabolic states in patients with coronary heart disease: a RCSCD-TCM study in China

**DOI:** 10.1186/s12933-022-01470-3

**Published:** 2022-03-11

**Authors:** Zhu Li, Yuanyuan He, Shuo Wang, Lin Li, Rongrong Yang, Yijia Liu, Qi Cheng, Lu Yu, Yanchao Zheng, Hongmei Zheng, Shan Gao, Chunquan Yu

**Affiliations:** 1 Tianjin University of Traditional Chinese Medicine, 10 Poyanghu Road, West Area, Tuanbo New Town, Jinghai District, Tianjin, 301617 China; 2 Tianjin Medical University General Hospital, 154 Anshan Road, Heping District, Tianjin, 300000 China

**Keywords:** Triglyceride, Fasting plasma glucose, TyG index, Coronary heart disease, Carotid plaques

## Abstract

**Background:**

The triglyceride glucose (TyG) index serves as a surrogate indicator of insulin resistance. However, there is limited evidence on the association between the TyG index and carotid artery plaque (CAP) in patients with coronary heart disease (CHD).

**Methods:**

The 10,535 CHD patients were divided according to TyG index quartiles (Q1: TyG index < 8.52; Q2: 8.52 ≤ TyG index < 8.93; Q3: 8.93 ≤ TyG index ≤ 9.40; Q4: TyG index > 9.40). The presence or absence of CAP was determined by carotid ultrasonography. Logistic regression was used to analyze the relationship between the TyG index and CAP in CHD patients. The relationship between the TyG index and CAP in according to sex, age groups, and glucose metabolism states were also assessed.

**Results:**

The baseline analysis showed that there were significant differences in related parameters among CHD patients divided into four groups according to the quartile of the TyG index. In the multi-adjusted modles, compared to Q1 of the TyG index, the odds ratios (OR) for Q4 of the TyG index for CAP were 1.37 (95% confidence interval [CI] 1.28–1.47) in CHD patients. The association between the TyG index and CAP in female (OR: 1.35; 95% CI 1.29–1.43) was higher than that in male (OR: 1.20; 95% CI 1.13–1.27). The OR value of middle-aged (≤ 60 years old) patients (OR: 1.34; 95% CI 1.26–1.42) was higher than that in elderly (> 60 years old) patients (OR: 1.16; 95% CI 1.11–1.22). In different glucose metabolism states, the TyG index of CHD patients was significantly related to the risk of CAP, with the highest OR value observed for diabetes (OR: 1.36; 95% CI 1.26–1.46).

**Conclusions:**

The TyG index and CAP showed a significant association in CHD patients. This association between TyG index and CAP in CHD patients is higher in female than in male, and the association in middle-aged and elderly patients is higher than that in elderly patients. In the condition of DM, the association between TyG index and carotid artery plaque in CHD patients is higher.

**Graphical abstract:**

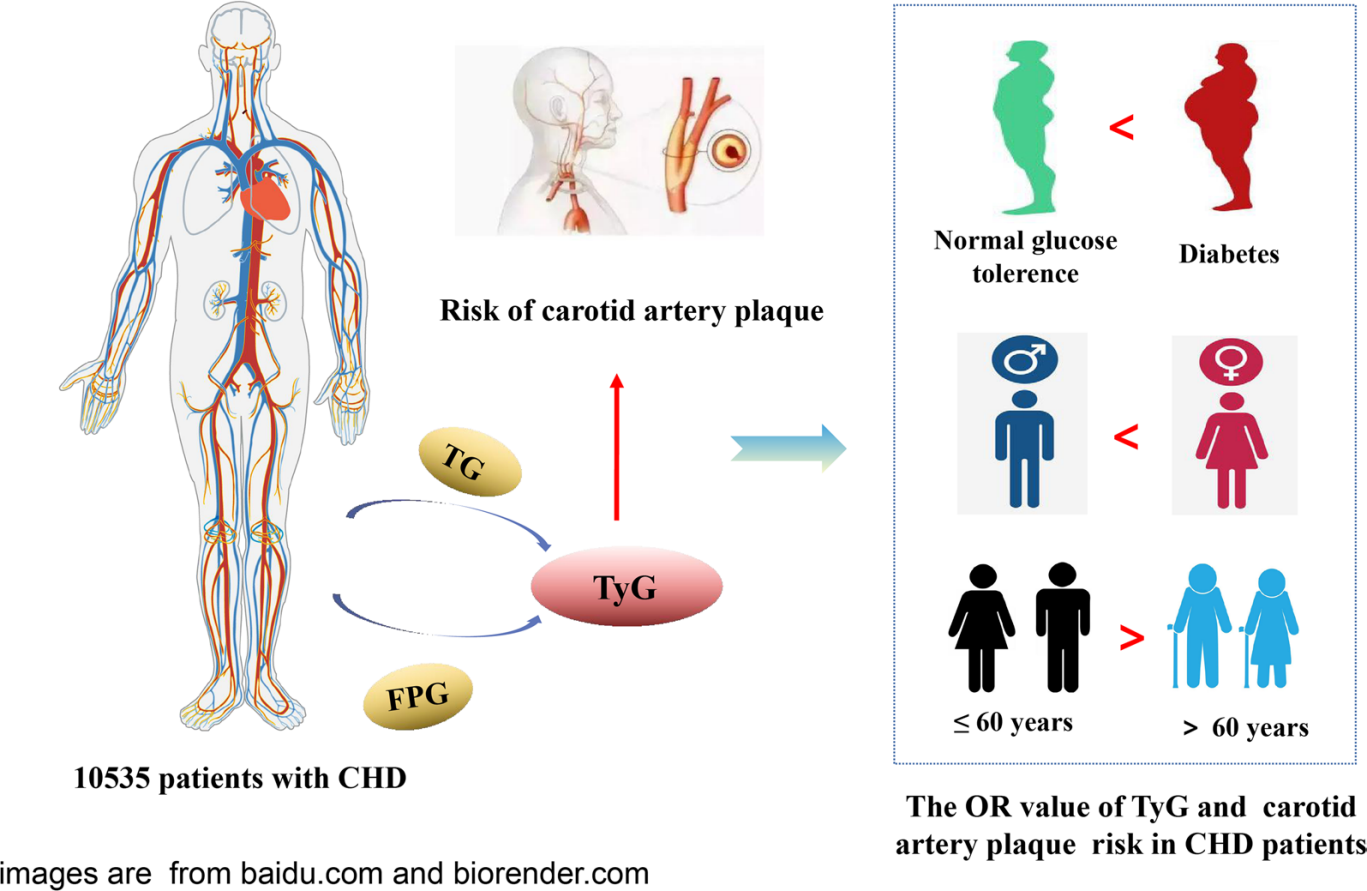

**Supplementary Information:**

The online version contains supplementary material available at 10.1186/s12933-022-01470-3.

## Background

Coronary heart disease (CHD) is a class of chronic noncommunicable diseases (NCD) with extremely high incidence and mortality rates. Diabetes mellitus (DM) usually coexists with arterial hypertension and dyslipidemia [1, 2]. Type 2 diabetes mellitus (T2DM) accounts for 95% of all the diseases diagnosed with DM. Which is one of the risk factors for coronary artery disease (CAD) and the progression and rupture of atherosclerotic plaques [3]. CAD is also a common comorbidity and major cause of death in DM patients. Studies have shown that in participants without CHD history, T2DM is associated with carotid artery plaques (CAP), which is a better predictor than high carotid artery intima-media thickness (CIMT) or recurrent cardiovascular events [4]. Pre-DM patients have a high propensity for developing DM [5]. Many studies have reported that CHD patients have a higher risk of adverse prognosis in Pre-DM and glucose metabolism disorders [6–8].

The triglyceride glucose (TyG) index is a valuable biomarker for the development of diabetes and is used as a marker of insulin resistance (IR), leading to the occurrence of NCD [9, 10]. The TyG index is related to the high prevalence of CAD, while the increased risk of major adverse cardiovascular and cerebrovascular events (MACEs) [11, 12], including ischemic stroke [13], increased arterial stiffness [14–16], hypertension [17], coronary artery stenosis [18], and carotid atherosclerosis (AS) [19] are related to the morbidity. However, no relevant studies have investigated the association between the TyG index and CAP in CHD patients according to their glucose metabolism statuses.

Therefore, this study aimed to clarify the association between the TyG index and CAP in the different glucose metabolic statuses of CHD patients, and to further investigate the association of TyG index and CAP in the different stratification of gender and age. In the clinical treatment of CHD, there is a need to identify simple biochemical indicators to prevent the risk of AS (such as CAP).

## Methods

### Patients

**Fig. 1:**
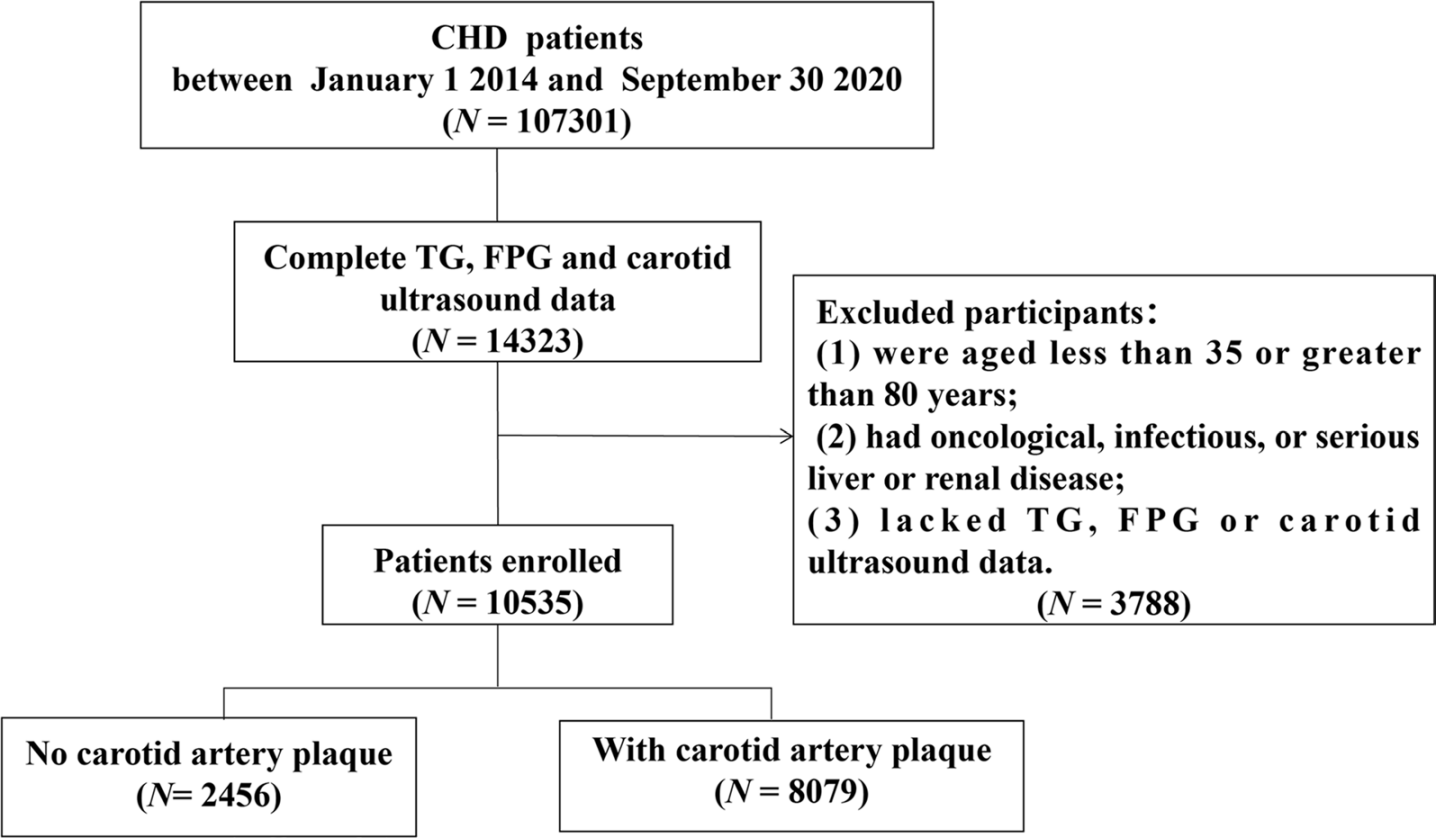
Flow chart of patient recruitment

This large-scale, multi-center retrospective cohort study included 107,301 CHD inpatients who were admitted to six hospitals in Tianjin between January 1, 2014, and September 30, 2020. The root investigation study design excluded patients aged less than 35 years or older than 80 years, patients with tumor, infectious, or severe liver or kidney disease, and patients lacking data on triglyceride (TG), fasting plasma glucose (FPG), and carotid ultrasound measurements. A total of 10,535 participants were eventually included in the study. A flowchart of the patients recruitment was shown in Fig. 1. This study was approved by the ethics committee of Tianjin University of Traditional Chinese Medicine (TJUTCM-EC20190008) and registered with the Chinese Clinical Trial Registry (ChiCTR-1900024535) and ClinicalTrials.gov (NCT04026724).

### Data collection

In this study, age, sex, smoking, drinking, and medication history of patients were recorded using standard structured questionnaires [20, 21]. Systolic blood pressure (SBP) and diastolic blood pressure (DBP) were measured by experienced technicians at the heart level using automatic blood pressure monitors. SBP ≥ 130 mmHg or DBP ≥ 80 mmHg was defined as hypertension [22].

Fasting venous blood samples were collected early in the morning from all participants. FPG, total cholesterol (TC), high-density lipoprotein cholesterol (HDL-C), TG, low-density lipoprotein cholesterol (LDL-C), C-reactionprotein (CRP), and glycated haemoglobin (HbA1c) leves were measured using an automatic haematology analyzer. Standard laboratory procedure for quality control were strictly followed [23]. The TyG index was calculated as Ln[fasting triglycerides (mg/dL) × fasting glucose (mg/dL)/2] [24]. Hyperlipidemia was defined as TC ≥ 6.2 mmol/L (240 mg/dL), TG ≥ 2.3 mmol/L (200 mg/dL), LDL-C ≥ 4.1 mmol/L (160 mg/dL), or HDL-C ≤ 1.0 mmol/L (40 mg/dL) [25]. Normal glucose tolerance (NGT) was defined as FPG < 5.6 mmol/L or HbA1c < 5.7%, Pre-DM was defined as FPG 5.6–6.9 mmol/L or HbA1c of 5.7–6.4%, DM was defined as FPG ≥ 7.0 mmol/L or HbA1c ≥ 6.5% [26].

The carotid ultrasound examinations were performed by a certified professional technician using a diagnostic ultrasound system. In B-mode imaging, the common carotid artery, internal carotid artery, and carotid artery bifurcation were scanned and imaged. CIMT was defined as the average IMT value of the left and right common carotid arteries [27]. Professional doctors analyzed the color of the carotid artery based on Doppler ultrasound results and recorded the number of CAPs and echo characteristics. CAP cases were divided into single (n = 1) and multiple (n ≥ 2). The echogenic properties of the CAP were categorized as hypoechoic, isoechoic, hyperechoic, and mixed types. This study employed rigorous quality control procedures to maintain consistency in the monitoring and test image acquisition and analysis. The inter-laboratory quality was assessed by licensed experimenters.

### Statistical analyses

The characteristics of the participants in the different groups were compared using *χ*^*2*^ tests and Kruskal–Wallis H tests. Odds ratios (ORs) and 95% confidence intervals (CIs) of CAP were estimated for the TyG index using logistic regression. Age, sex, SBP, DBP, CRP, HbA1c, TC, HDL-C, LDL-C, smoking, drinking, hypertension, hyperlipidemia, use of antihypertensives, and use of antilipidemic were considered as potential confounders in this association. The collinearity of the different models was tested before logistic regression. Missing values were imputed using the multiple imputation method. All statistical analyses were performed using SPSS 24.0 (IBM Corp, New York, NY, USA).

## Results

### Baseline characteristics

The basic characteristics of the study population of 10,535 cases are shown in Table 1. The median age of the participants was 64 years old, and the proportion of female (51.4%) was slightly higher than that of male (48.6%). Among them, 8079 (76.69%) patients had CAP, while the highest proportion of DM patients was 37.6%. The subjects were divided into four groups according to the quartile level of the TyG index. Generally speaking, DBP, SBP, FPG, TC, TG, HbA1c, smoking, drinking, hyperlipidemia, use of antihypertensives, and use of antilipidemic were positively associated with the quartile level of the TyG index, while HDL-C was negatively associated with the quartile level of the TyG index.

**Table 1:** General characteristics of the study participants according to the TyG index

Characteristic	Total (*N* = 10,535)	TyG index				*P-*value
		Q1 (*n* = 2650)	Q2 (*n* = 2647)	Q3 (*n* = 2590)	Q4 (*n* = 2648)	
Sex, n (%)						< 0.001
Male	5121 (48.6)	1375 (51.9)	1256 (47.4)	1183 (45.7)	1307 (49.4)	
Female	5414 (51.4)	1275 (48.1)	1391 (52.6)	1407 (54.3)	1341 (50.6)	
Age, years, median (IQR)						< 0.001
Total	64.0 (59.0–70.0)	65.0 (60.0–71.0)	64.0 (59.0–69.0)	64.0 (59.0–69.0)	63.0 (57.0–68.0)	
≤ 60	55.0 (51.0–58.0)	55.0 (51.0–58.0)	55.0 (52.0–58.0)	55.0 (52.0–58.0)	55.0 (51.0–57.0)	
> 60	67.0 (64.0–71.0)	68.0 (64.0–72.0)	67.0 (64.0–71.0)	67.0 (63.0–71.0)	67.0 (64.0–71.0)	
SBP, mmHg, median (IQR)	140.0 (128.0–156.0)	139.0 (124.0–152.0)	140.0 (126.0–155.0)	140.0 (130.0–159.0)	141.0 (130.0–159.0)	< 0.001
DBP, mmHg, median (IQR)	83.0 (77.0–90.0)	80.0 (75.0–90.0)	82.0 (76.0–90.0)	83.0 (78.0–90.0)	84.0 (78.0–91.0)	< 0.001
CRP, mg/L, median (IQR)	4.2 (2.0–12.8)	4.1 (1.9–14.9)	4.3 (2.0–14.2)	4.0 (2.0–11.3)	4.5 (2.2–11.2)	< 0.001
FPG, mmol/L, median (IQR)	6.2 (5.3–8.1)	5.3 (4.8–5.9)	5.8 (5.1–6.8)	6.54 (5.6–8.1)	9.4 (7.0–12.6)	< 0.001
LDL-C, mmol/L, median (IQR)	2.8 (2.1–3.4)	2.5 (1.9–3.1)	2.8 (2.2–3.4)	3.0 (2.3–3.6)	2.8 (2.2–3.5)	< 0.001
HDL-C, mmol/L, median (IQR)	1.1 (0.9–1.3)	1.2 (1.0–1.4)	1.1 (0.9–1.3)	1.1 (0.9–1.2)	1.0 (0.8–1.1)	< 0.001
TG, mmol/L, median (IQR)	1.4 (1.0–2.1)	0.9 (0.7–1.0)	1.3 (1.1–1.5)	1.8 (1.5–2.1)	2.6 (2.0–3.5)	< 0.001
TC, mmol/L, median (IQR)	4.6 (3.8–5.4)	4.1 (3.4–4.9)	4.5 (3.8–5.2)	4.7 (4.0–5.5)	5.0 (4.2–5.7)	< 0.001
HbA1c, mmol/L, median (IQR)	6.0 (5.6–7.0)	5.7 (5.4–6.2)	5.9 (5.5–6.5)	6.1 (5.6–6.9)	7.1 (6.1–8.6)	< 0.001
TyG index	9.0 (8.5–9.4)	8.2 (8.0–8.4)	8.7 (8.6–8.8)	9.1 (9.0–9.3)	9.8 (9.6–10.2)	< 0.001
Smoking, n (%)	4590 (43.6)	1173 (44.3)	1121 (42.3)	1101 (42.5)	1195 (45.1)	< 0.001
Drinking, n (%)	5863 (55.7)	1457 (55)	1480 (55.9)	1421 (54.9)	1505 (56.8)	< 0.001
Hypertension, n (%)	8875 (84.2)	2114 (23.8)	2201 (24.8)	2241(25.3)	2319 (26.1)	< 0.001
Hyperlipidemia, n (%)	5770 (54.8)	690 (12.0)	961 (16.7)	1326 (23.0)	1981 (34.3)	< 0.001
Use of antihypertensives, n (%)	5239 (49.7)	1160 (43.8)	1240 (46.8)	1363 (52.6)	1476 (55.7)	< 0.001
Use of antilipidemic, n (%)	7787 (73.9)	1703 (64.3)	1947 (73.6)	2026 (78.2)	2111 (79.7)	< 0.001
CIMT, mm, median (IQR)	0.10 (0.09–0.12)	0.10 (0.09–0.12)	0.10 (0.09- 0.12)	0.10 (0.09–0.12)	0.10 (0.09–0.12)	0.742
Carotid artery plaque, n (%)	8079 (76.69)	1985 (74.91)	1996 (75.41)	2006 (77.45)	2092 (79.00)	< 0.001
Glucose regulation state, n (%)						< 0.001
Normal glucose regulation	3756 (35.7)	1735 (65.5)	1160 (43.8)	654 (25.3)	207 (7.8)	
Prediabetes	2813 (26.7)	694 (26.2)	861 (32.5)	834 (32.2)	424 (16.0)	
Diabetes	3966 (37.6)	221 (8.3)	626 (23.7)	1102 (42.5)	2017 (76.2)	
No. of carotid artery plaque, n (%)						< 0.001
0	2456 (23.3)	665 (25.1)	651 (24.6)	584 (22.5)	556 (21)	
1	406 (3.9)	96 (3.6)	100 (3.8)	100 (3.9)	110 (4.2)	
≥ 2	7673 (72.8)	1889 (71.3)	1896 (71.6)	1906 (73.6)	1982 (74.8)	
Carotid artery plaque echo property, n (%)						< 0.001
Hypoechoic plaque	497 (4.7)	114 (4.3)	131 (4.9)	130 (5)	122 (4.6)	
Isoechoic plaque	548 (5.2)	137 (5.2)	133 (5)	136 (5.3)	142 (5.4)	
Hyperechoic plaque	4792 (45.5)	1211 (45.7)	1162 (43.9)	1194 (46.1)	1225 (46.3)	
Mixture plaque	2242 (21.3)	523 (19.7)	570 (21.5)	546 (21.1)	603 (22.8)	

Data are presented as median (interquartile) or number (proportion, %)

Q1: TyG index < 8.52, Q2: 8.52 ≤ TyG index < 8.93, Q2: 8.93 ≤ TyG index ≤ 9.40, Q4: TyG index > 9.40

TyG, triglyceride-glucose; SBP, systolic blood pressure; DBP, diastolic blood pressure; CRP, C-reactionprotein; FPG, fasting plasma glucose; TC, total cholesterol; TG, triglycerides; HDL-C, high-density lipoprotein cholesterol; LDL-C, low-density lipoprotein cholesterol; HbA1c, glycated haemoglobin; IQR, interquartile range

### Association between the TyG index and the risk of carotid artery plaques

As shown in Table 2, in multivariate logistic regression analysis, when the TyG index was used as a continuous variable, it was significantly associated with the risk of CAP (OR: 1.29; 95% CI 1.24–1.34). The association between the TyG index and CAP was further explored using the TyG index as a categorical variable. Multivariate logistic regression analysis showed that the TyG index levels for Q3 and Q4 were associated with an increased OR for CAP when Q1 was used as a reference, with the highest association observed for Q4 (OR: 1.37; 95% CI 1.28–1.47). In both unadjusted and adjusted models, the TyG index (quartiles) was consistent with the *P* for trend of the CAP, when the TyG index was served as a continuous variable (*P* < 0.001). The association of TyG index with the number and echoproperties of carotid plaques were further evaluated. The results show that the association remained significant (Adittional file 1: Table S1–S2).

**Table 2:** Association between the TyG index and the risk of carotid artery plaques

Variables	Carotid artery plaques					
	OR (95% CI)^a^	*P-*value	OR (95% CI)^b^	*P-*value	OR (95% CI)^c^	*P-*value
TyG index	1.14 (1.11–1.17)	< 0.001	1.28 (1.24–1.31)	< 0.001	1.29 (1.24–1.34)	< 0.001
Q1	Reference		Reference		Reference	
Q2	1.00 (0.95–1.05)	0.969	1.10 (1.04–1.16)	0.537	1.01 (0.96–1.07)	0.682
Q3	1.09 (1.04–1.15)	0.001	1.22 (1.16–1.29)	0.013	1.07 (1.01–1.14)	0.025
Q4	1.22 (1.16–1.28)	< 0.001	1.51 (1.43–1.59)	< 0.001	1.37 (1.28–1.47)	< 0.001
*P-*trend		< 0.001		< 0.001		< 0.001

^a^Model 1: unadjusted

^b^Model 2: adjusted for sex, age, SBP, DBP

^c^Model 3: adjusted for sex, age, SBP, DBP, CRP, TC, HDL-C, LDL-C, smoking, drinking, hypertension, hyperlipidemia, use of antihypertensives, and use of antilipidemic

Regardless of sex, this relationship remained statistically significant after adjusting for variables. As shown in Table 3, after multivariate adjustment, the association between the TyG index and CAP in female (OR: 1.35; 95% CI 1.29–1.43), which was higher than that in male (OR: 1.20; 95% CI 1.13–1.27). As shown in Table 4, after multivariate adjustment, the TyG index of CHD patients was significantly associated with CAP at different ages. The OR value of middle-aged (≤ 60 years old) patients (OR: 1.34; 95% CI 1.26–1.42) was higher than that of elderly (> 60 years old) patients (OR: 1.16; 95% CI 1.11–1.22). For both sexes and different ages, using Q1 as the reference, Q4 was significantly related to the increased risks of CAP, even after multivariate adjustment, this relationship remained significant.

**Table 3:** Association between the TyG index and the risk of carotid artery plaques according to sex

Sex	Variables	Carotid artery plaques					
		OR (95% CI)^a^	*P-*value	OR (95% CI)^b^	*P-*value	OR (95% CI)^c^	*P-*value
Male	TyG index	0.94 (0.91–0.98)	0.003	1.14 (1.10–1.19)	< 0.001	1.20 (1.13–1.27)	< 0.001
	Q1	Reference		Reference		Reference	
	Q2	1.00 (0.91–1.07)	0.789	1.13 (1.04–1.23)	0.004	1.05 (0.96–1.15)	0.268
	Q3	0.95 (0.88–1.03)	0.213	1.17 (1.07–1.28)	< 0.001	1.03 (0.94–1.13)	0.548
	Q4	0.87 (0.80–0.94)	< 0.001	1.23 (1.13–1.34)	< 0.001	1.18 (1.06–1.31)	0.003
Female	TyG index	1.34 (1.30–1.39)	< 0.001	1.38 (1.33–1.44)	< 0.001	1.35 (1.29–1.43)	< 0.001
	Q1	Reference		Reference		Reference	
	Q2	1.08 (1.01–1.15)	0.019	1.08 (1.01–1.16)	0.031	0.99 (0.92–1.07)	0.875
	Q3	1.31 (1.23–1.40)	< 0.001	1.25 (1.17–1.34)	< 0.001	1.10 (1.01–1.19)	0.019
	Q4	1.62 (1.52–1.74)	< 0.001	1.74 (1.61–1.87)	< 0.001	1.52 (1.38–1.66)	< 0.001

^a^Model 1: unadjusted

^b^Model 2: adjusted for age, SBP, DBP

^c^Model 3: adjusted for age, SBP, DBP, CRP, TC, HDL-C, LDL-C, smoking, drinking, hypertension, hyperlipidemia, use of antihypertensives, and use of antilipidemic

**Table 4:** Association between the TyG index and the risk of carotid artery plaques according to age

Age	Variables	Carotid artery plaques					
		OR (95% CI)^a^	*P-*value	OR (95% CI)^b^	*P-*value	OR (95% CI)^c^	*P-*value
≤ 60	TyG index	1.37 (1.32–1.42)	< 0.001	1.27 (1.22–1.32)	< 0.001	1.34 (1.26–1.42)	< 0.001
	Q1	Reference		Reference		Reference	
	Q2	1.19 (1.09–1.29)	< 0.001	1.18 (1.08–1.30)	< 0.001	1.05(0.95–1.16)	0.359
	Q3	1.59 (1.45–1.73)	< 0.001	1.45 (1.32–1.59)	< 0.001	1.23 (1.11–1.37)	< 0.001
	Q4	1.90 (1.75–2.06)	< 0.001	1.68 (1.54–1.83)	< 0.001	1.54 (1.37–1.72)	< 0.001
> 60	TyG index	1.16 (1.12–1.21)	< 0.001	1.21 (1.16–1.25)	< 0.001	1.16 (1.11–1.22)	< 0.001
	Q1	Reference		Reference		Reference	
	Q2	0.99 (0.93–1.06)	0.840	1.05 (1.00–1.12)	0.170	0.98 (0.91–1.05)	0.563
	Q3	0.99 (0.93–1.05)	0.720	1.07 (1.00–1.15)	0.040	0.95 (0.88–1.02)	0.161
	Q4	1.23 (1.15–1.32)	< 0.001	1.30 (1.21–1.39)	< 0.001	1.15 (1.06–1.26)	0.002

^a^Model 1: unadjusted

^b^Model 2: adjusted for sex, SBP, DBP

^c^Model 3: adjusted for sex, SBP, DBP, CRP, TC, HDL-C, LDL-C, smoking, drinking, hypertension, hyperlipidemia, use of antihypertensives, and use of antilipidemic

### Association between the TyG index and CAP according to glucose regulation state

As shown in Table 5, after multivariate adjustment showed significant associations between the TyG index and the risk of CAP in CHD patients according to different glucose metabolism states, with the highest OR value observed for DM (OR: 1.36; 95% CI 1.26–1.46). Taking the Q1 as a reference, Q4 was significantly associated with an increased risk of CAP during the DM.

**Table 5:** Association between TyG index and the risk of carotid artery plaques according to glucose regulation state

Glucose regulation state	Variables	Carotid artery plaques					
		OR (95% CI)^a^	*P*-value	OR (95% CI)^b^	*P-*value	OR (95% CI)^c^	*P*-value
Normal glucose regulation	TyG index	0.95 (0.90–1.01)	0.080	1.16 (1.09–1.24)	< 0.001	1.11 (1.02–1.22)	0.017
	Q1	Reference		Reference		Reference	
	Q2	0.98 (0.92–1.05)	0.526	1.11 (1.03–1.19)	0.007	1.04 (0.95–1.13)	0.412
	Q3	0.94 (0.86–1.02)	0.112	1.18 (1.08–1.29)	< 0.001	1.03 (0.93–1.15)	0.563
	Q4	0.81 (0.71–0.92)	0.001	1.11 (0.97–1.28)	0.129	1.11 (0.93–1.32)	0.259
Prediabetes	TyG index	0.87 (0.82–0.92)	< 0.001	1.06 (0.99–1.14)	0.078	1.08 (0.97–1.19)	0.182
	Q1	Reference		Reference		Reference	
	Q2	0.90 (0.83–0.99)	0.032	1.06 (0.96–1.17)	0.288	0.94 (0.85–1.05)	0.295
	Q3	1.02 (0.93–1.12)	0.645	1.23 (1.11–1.37)	< 0.001	1.06 (0.94–1.20)	0.330
	Q4	0.69 (0.62–0.77)	< 0.001	0.99 (0.88–1.12)	0.879	0.90 (0.76–1.06)	0.202
Diabetes	TyG index	1.16 (1.11–1.22)	< 0.001	1.36 (1.30–1.43)	< 0.001	1.36 (1.26–1.46)	< 0.001
	Q1	Reference		Reference		Reference	
	Q2	1.11 (0.97–1.28)	0.142	1.26 (1.09–1.47)	0.003	1.13 (0.96–1.33)	0.147
	Q3	1.18 (1.03–1.35)	0.017	1.37 (1.19–1.58)	< 0.001	1.07 (0.91–1.25)	0.408
	Q4	1.39 (1.22–1.58)	< 0.001	1.87 (1.63–2.15)	< 0.001	1.42 (1.21–1.66)	< 0.001

^a^Model 1: unadjusted

^b^Model 2: adjusted for age, sex, SBP, DBP

^c^Model 3: adjusted for age, sex, SBP, DBP, CRP, TC, HDL-C, LDL-C, smoking, drinking, hypertension, hyperlipidemia, use of antihypertensives, and use of antilipidemic

This study also observed a significant association between TyG index and CAP risk in male patients with NGT (OR: 1.21; 95% CI 1.06–1.39). The association in females (OR: 1.45; 95% CI 1.32–1.60) with DM status was higher than in males (OR: 1.23; 95% CI 1.10–1.37) (Table 6). The association with CAP in CHD patients with DM status aged > 60 years old (OR: 1.35; 95% CI 1.24–1.48) with DM status was higher than those aged ≤ 60 years old (OR: 1.21; 95% CI 1.08–1.35) (Table 7).

**Table 6:** Association between the TyG index and the risk of carotid artery plaques according to different glucose regulation state and sex

Sex	Glucose regulation state	Variables	Carotid artery plaques					
			OR (95% CI)^a^	*P-*value	OR (95% CI)^b^	*P-*value	OR (95% CI)^c^	*P-*value
Male	Normal glucose regulation	TyG index	0.88 (0.81–0.96)	0.005	1.24 (1.13–1.36)	< 0.001	1.21 (1.06–1.39)	0.005
		Q1	Reference		Reference		Reference	
		Q2	0.98 (0.88–1.10)	0.751	1.23 (1.09–1.38)	0.001	1.06 (0.93–1.21)	0.398
		Q3	0.79 (0.69–0.90)	< 0.001	1.10 (0.96–1.27)	0.182	0.83 (0.70–0.96)	0.033
		Q4	0.57 (0.48–0.68)	< 0.001	1.00 (0.82–1.22)	0.974	0.91 (0.69–1.19)	0.494
	Prediabetes	TyG index	0.67 (0.61–0.74)	< 0.001	0.97 (0.87–1.07)	0.505	0.95 (0.82–1.12)	0.557
		Q1	Reference		Reference		Reference	
		Q2	0.94 (0.81–1.10)	0.420	1.05 (0.89–1.24)	0.552	0.94 (0.79–1.12)	0.511
		Q3	0.85 (0.73–0.99)	0.038	1.18 (1.00–1.39)	0.055	1.02 (0.84–1.23)	0.846
		Q4	0.49 (0.42–0.58)	< 0.001	0.83 (0.69–1.00)	0.045	0.67 (0.52–0.86)	0.002
	Diabetes	TyG index	0.91 (0.84–0.97)	0.005	1.11 (1.03–1.19)	0.009	1.23 (1.10–1.37)	< 0.001
		Q1	Reference		Reference		Reference	
		Q2	0.99 (0.78–1.24)	0.904	1.19 (0.94–1.51)	0.143	1.13 (0.88–1.45)	0.331
		Q3	1.06 (0.85–1.32)	0.592	1.34 (1.07–1.67)	0.011	1.13 (0.90–1.44)	0.284
		Q4	0.98 (0.79–1.20)	0.821	1.48 (1.19–1.84)	< 0.001	1.37 (1.08–1.74)	0.010
Female	Normal glucose regulation	TyG index	1.05 (0.97–1.14)	0.198	1.09 (1.00–1.19)	0.046	1.02 (0.90–1.15)	0.794
		Q1	Reference		Reference		Reference	
		Q2	1.06 (0.97–1.15)	0.223	1.04 (0.94–1.14)	0.469	1.01 (0.91–1.23)	0.825
		Q3	1.17 (1.06–1.30)	0.003	1.22 (1.09–1.37)	0.001	1.16 (1.01–1.33)	0.043
		Q4	1.03 (0.86–1.24)	0.739	1.23 (1.01–1.5)	0.041	1.22 (0.95–1.56)	0.115
	Prediabetes	TyG index	1.05 (0.97–1.14)	0.244	1.14 (1.04–1.24)	0.005	1.17 (1.02–1.35)	0.023
		Q1	Reference		Reference		Reference	
		Q2	0.94 (0.84–1.06)	0.327	1.05 (0.93–1.20)	0.410	0.93 (0.81–1.07)	0.336
		Q3	1.22 (1.08–1.38)	0.001	1.26 (1.11–1.44)	< 0.001	1.10 (0.94–1.29)	0.222
		Q4	0.89 (0.77–1.03)	0.127	1.12 (0.96–1.31)	0.145	1.12 (0.89–1.40)	0.328
	Diabetes	TyG index	1.44 (1.35–1.53)	< 0.001	1.58 (1.48–1.69)	< 0.001	1.45 (1.32–1.60)	< 0.001
		Q1	Reference		Reference		Reference	
		Q2	1.31 (1.08–1.59)	0.005	1.30 (1.06–1.59)	0.012	1.14 (0.91–1.42)	0.261
		Q3	1.44 (1.21–1.73)	< 0.001	1.38 (1.14–1.67)	0.001	1.01 (0.82–1.25)	0.930
		Q4	1.97 (1.65–2.34)	< 0.001	2.15 (1.78–2.58)	< 0.001	1.42 (1.14–1.77)	0.002

^a^Model 1: unadjusted

^b^Model 2: adjusted for age, SBP, DBP

^c^Model 3: adjusted for age, SBP, DBP, CRP, TC, HDL-C, LDL-C, smoking, drinking, hypertension, hyperlipidemia, use of antihypertensives, and use of antilipidemic

**Table 7:** Association between the TyG index and the risk of carotid artery plaques according to different glucose regulation state and age

Age	Glucose regulation state	Variables	carotid artery plaques					
			OR (95% CI)^a^	*P-*value	OR (95% CI)^b^	*P-*value	OR (95% CI)^c^	*P-*value
≤ 60	Normal glucose regulation	TyG index	1.32 (1.22–1.44)	< 0.001	1.16 (1.06–1.28)	< 0.001	1.12 (0.98–1.28)	0.099
		Q1	Reference		Reference		Reference	
		Q2	1.27 (1.14–1.43)	< 0.001	1.24 (1.10–1.40)	< 0.001	1.17 (1.02–1.33)	0.026
		Q3	1.53 (1.34–1.74)	< 0.001	1.40 (1.22–1.60)	< 0.001	1.24 (1.04–1.46)	0.015
		Q4	1.38 (1.15–1.64)	< 0.001	1.09 (0.91–1.32)	0.360	1.11 (0.86–1.43)	0.430
	Prediabetes	TyG index	1.05 (0.96–1.15)	0.290	0.99 (0.90–1.09)	0.770	0.93 (0.80–1.09)	0.367
		Q1	Reference		Reference		Reference	
		Q2	0.86 (0.73–1.02)	0.090	0.92 (0.77–1.09)	0.320	0.74 (0.61–0.90)	0.002
		Q3	1.21 (1.02–1.43)	0.030	1.12 (0.94–1.34)	0.190	0.86 (0.70–1.05)	0.141
		Q4	1.08 (0.91–1.29)	0.390	1.04 (0.87–1.26)	0.650	0.89 (0.69–1.15)	0.374
	Diabetes	TyG index	1.21 (1.13–1.30)	< 0.001	1.16 (1.08–1.25)	< 0.001	1.21(1.08–1.35)	0.001
		Q1	Reference		Reference		Reference	
		Q2	1.32 (0.98–1.78)	0.070	1.23 (0.91–1.67)	0.190	0.90 (0.64–1.26)	0.544
		Q3	1.71 (1.29–2.27)	< 0.001	1.49 (1.11–2.00)	0.010	0.95 (0.68–1.31)	0.738
		Q4	1.98 (1.51–2.60)	< 0.001	1.69 (1.27–2.24)	< 0.001	1.03 (0.74–1.43)	0.866
> 60	Normal glucose regulation	TyG index	0.94 (0.87–1.02)	0.160	1.05 (0.96–1.14)	0.300	0.99 (0.88–1.11)	0.817
		Q1	Reference		Reference		Reference	
		Q2	0.94 (0.86–1.03)	0.200	1.01 (0.92–1.11)	0.780	0.94 (0.85–1.04)	0.241
		Q3	0.85 (0.76–0.95)	< 0.001	0.99 (0.88–1.11)	0.890	0.87 (0.76–1.00)	0.048
		Q4	0.90 (0.74–1.10)	0.320	0.95 (0.77–1.17)	0.640	0.90 (0.70–1.16)	0.420
	Prediabetes	TyG index	0.98 (0.90–1.07)	0.640	1.03 (0.94–1.13)	0.510	0.96 (0.84–1.10)	0.568
		Q1	Reference		Reference		Reference	
		Q2	1.03 (0.91–1.15)	0.680	1.08 (0.96–1.21)	0.230	0.97 (0.85–1.11)	0.686
		Q3	1.09 (0.97–1.23)	0.150	1.19 (1.05–1.35)	0.010	1.01 (0.87–1.17)	0.916
		Q4	0.76 (0.66–0.88)	< 0.001	0.80 (0.69–0.94)	0.010	0.65 (0.52–0.80)	< 0.001
	Diabetes	TyG index	1.38 (1.29–1.47)	< 0.001	1.43 (1.34–1.53)	< 0.001	1.35 (1.24–1.48)	< 0.001
		Q1	Reference		Reference		Reference	
		Q2	1.18 (0.99–1.40)	0.060	1.25 (1.05–1.49)	0.010	1.19 (0.99–1.43)	0.059
		Q3	1.15 (0.98–1.35)	0.080	1.25 (1.06–1.47)	0.010	1.05 (0.88–1.25)	0.618
		Q4	1.63 (1.39–1.90)	< 0.001	1.78 (1.52–2.09)	< 0.001	1.45 (1.21–1.74)	< 0.001

^a^Model 1: unadjusted

^b^Model 2: adjusted for sex, SBP, DBP

^c^Model 3: adjusted for sex, SBP, DBP, CRP, TC, HDL-C, LDL-C, smoking, drinking, hypertension, hyperlipidemia, use of antihypertensives, and use of antilipidemic

## Discussion

The results of this study revealed a significant association between the TyG index and CAP in CHD patients. This is the first large-scale study to demonstrate this relationship between the TyG index and CAP in CHD patients, and assessed this relationship according to sex, ages and glucose metabolism states.

Studies in recent years have shown the close relationship between the TyG index and the homeostasis model assessment of insulin resistance (HOMA-IR). And the predictive value of the TyG index for IR was better than that for HOMA-IR [19]. Therefore, the TyG index reflect the indicator of peripheral IR. A cross-sectional study reported that the TyG index was positively associated with the prevalence of CAD and could be used as a marker of AS [28]. Compared to patients with the lowest the TyG index, those in the quartile with the highest TyG index have a higher risk of stroke and myocardial infarction (MI) [29]. The TyG index was also significantly associated with the progression of arterial stiffness in hypertensive people but not prehypertensive individuals [30]. The TyG index was also closely related to coronary artery calcification and carotid AS [31]. This finding is consistent with the results of the present study. The related mechanism of action may involve several aspects. Firstly, insulin can cause lipid hyaluronic degeneration by enhancing sympathetic nerve activity or acting as a growth factor. Lipohyaline deposition can block small arteries, leading to the development of CVD [32]. Secondly, the TyG index is associated with inflammation. IR can induce inflammation, oxidative stress, and metabolic changes, causing damage to the vascular endothelium due to inflammation [33]. Therefore, the TyG index in CHD patients was associated with the occurrence of CAP, in which a high level of TyG index was associated with the occurrence of higher CAP.

The close association between the TyG index and cerebral small vessel disease (cSVD) may be caused by other concomitant metabolic syndromes. IR patients usually have other comorbidities, including hypertension, DM and obesity [13, 34]. The TyG index has received attention in the field of DM and metabolism, and has a positive impact on the assessment and prediction of IR and metabolic syndrome in DM patients. A higher TyG index was associated with an increased risk of coronary artery stenosis in asymptomatic T2DM patients [35]. However, cardiometabolic heterogeneity in non-DM individuals has been reported [36, 37]. Consistent with the results of this study, this study observed a significant association between the TyG index in CHD patients and the risk of CAP according to glucose metabolism states and after adjusting for confounding variables, with the highest OR value observed for DM.

Recent studies have shown that female have a lower risk of cardiovascular disease compared to male, but that hyperglycemia and hyperinsulinemia caused by IR may reverse this sex-based protection. All insulin replacement markers showed that good association with HOMA-IR of both sexes, and the association between female and HOMA-IR was stronger than that of male [38]. However, some studies have found that there was no sex difference between the TyG index and MACEs in patients with hypertension [39]. There has been an investigation of the association between the TyG index and the early stages of subclinical atherosclerosis (SA) between the sexes. A high TyG index was independently associated with SA in non-diabetic female, but in NGT male. Regardless of sex, the TyG index is unrelated to the presence of SA in DM patients [40]. Higher TG and blood pressure had greater impact in both DM patients and those with NGT. Moreover, the number of CVD events and deaths was higher in female than that in male [41]. Consistent with the results of this study, the TyG index in female was more highly associated with CAP compared to the association in male. A high TyG index showed a higher association with CAP.

In the middle-aged and elderly populations, an increase in the TyG index was significantly associated with hypertension and isolated systolic hypertension [42]. This study showed that the OR value of middle-aged patients with CHD was higher than that of elderly patients with CHD. The TyG index was significantly related to the risk of CAP in middle-aged patients with NGT, whereas the TyG index was significantly related to the risk of CAP in elderly patients with DM. This may be because this study is a CHD population, with an average age of over 60 years old, belonging to the middle-aged and elderly population, and there is a certain bias for age. Therefore, future research should inclued people of different ages to determine the association between the TyG index and CAP according to ages.

To sum up, with an increasing number of studies on the influence of the TyG index on patients with cardiovascular diseases, the clinical significance of the TyG index is becoming increasingly clear. Evaluation of the TyG index may have important clinical significance for risk stratification and individualized treatment of CHD patients.

## Strengths and limitations

This large-scale, multi-center cohort study had several limitations. First, this study was a multi-center study, thus, there may be bias in the measurement methods at different research centers. However, the practitioners conducted external quality assessments between clinical laboratories in each center. Second, this study was a cross-sectional study. Therefore, the results of this study cannot establish causality. The exact mechanism of the relationship between the TyG index and CAP requires further prospective large-scale research.

## Conclusion

This study demonstrated a significant association between the TyG index and CAP in CHD patients. In addition, the association between the TyG index and CAP in CHD patients was higher in female than in male, and higher in middle-aged and elderly than in the elderly. In DM patients, the association between the TyG index and CAP in CHD patients was higher. As a marker of IR, the TyG index is easy to calculate and may reflect the risk of CAP in CHD patients. The results of this study may emphasize the need for a risk management strategy for specific sex and different age groups to prevent the occurrence of CAP in CHD patients.

## Supplementary Information

**Additional file 1: Table S1.** Correlation between the TyG index and the number of carotid artery plaque. **Table S2. **Correlation between the TyG index and the echogenicity of carotid artery plaque.

## Data Availability

The datasets used and/or analyzed in the current study are available from the corresponding author upon reasonable request.

## Abbreviations

TyG: Triglyceride glucose
CHD: Coronary heart disease
DM: Diabetes mellitus
NCD: Non-communicable diseases
T2DM: Type 2 diabetes
CAD: Coronary artery disease
CAP: Carotid artery plaque
CIMT: Carotid artery intima-media thickness
Pre-DM: Prediabetes
AS: Atherosclerosis
SBP: Systolic blood pressure
DBP: Diastolic blood pressure
FPG: Fasting plasma glucose
TC: Total cholesterol
HDL-C: High-density lipoprotein cholesterol
TG: Triglycerides
LDL-C: Low-density lipoprotein cholesterol
CRP: C-reactionprotein
HbA1c: Glycated haemoglobin
OR: Odds ratios
CIs: Confidence intervals
HOMA-IR: Homeostasis model assessment of insulin resistance
SA: Subclinical atherosclerosis
