# Supplementary material for: Association between triglyceride glucose index and carotid artery plaque in different glucose metabolic states in patients with coronary heart disease: a RCSCD-TCM study in China
Source: Cardiovasc Diabetol. 2022 Mar 11;21:38. doi: 10.1186/s12933-022-01470-3 (PMC8917731; doi:10.1186/s12933-022-01470-3)
Supplement: Supplementary file 1 — Additional file 1: Table S1. Correlation between the TyG index and the number of carotid artery plaque. Table S2. Correlation between the TyG index and the echogenicity of carotid artery plaque. [file 12933_2022_1470_MOESM1_ESM.docx]

**Table S1. Correlation between the TyG index and the number of carotid artery plaque**

| Number of carotid artery plaque | Variables | OR (95 %CI)^a^ | *P-*value | OR (95 %CI)^b^ | *P-*value | OR (95 %CI)^c^ | *P-*value |
| --- | --- | --- | --- | --- | --- | --- | --- |
| 1 | TyG index | 1.12 (1.07-1.17) | < 0.001 | 1.16 (1.10-1.21) | < 0.001 | 1.25 (1.17-1.33) | < 0.001 |
|  | Q1 | 0.82 (0.77-0.89) | < 0.001 | 0.79 (0.73-0.86) | < 0.001 | 0.84 (0.77-0.92) | < 0.001 |
|  | Q2 | 0.94 (0.88-1.02) | 0.120 | 0.94 (0.88-1.01) | 0.114 | 0.92 (0.86-1.00) | 0.041 |
|  | Q3 | 1.12 (1.04-1.21) | 0.003 | 1.11 (1.04-1.20) | 0.004 | 1.05 (0.98-1.13) | 0.193 |
|  | Q4 | 1.15 (1.07-1.24) | < 0.001 | 1.20 (1.11-1.29) | < 0.001 | 1.27(1.17-1.39) | < 0.001 |
| ≥2 | TyG index | 1.12 (1.10-1.15) | < 0.001 | 1.26 (1.22-1.29) | < 0.001 | 1.69 (1.63-1.75) | < 0.001 |
|  | Q1 | 0.91 (0.87-0.95) | < 0.001 | 0.80 (0.76-0.83) | < 0.001 | 0.76 (0.72-0.80) | < 0.001 |
|  | Q2 | 0.91 (0.87-0.95) | < 0.001 | 0.90 (0.86-0.94) | < 0.001 | 0.86 (0.82-0.90) | < 0.001 |
|  | Q3 | 1.04 (1.00-1.09) | 0.039 | 1.06 (1.01-1.10) | 0.013 | 0.99 (0.95-1.04) | 0.708 |
|  | Q4 | 1.16 (1.12-1.21) | < 0.001 | 1.33 (1.28-1.39) | < 0.001 | 1.69 (1.60-1.78) | < 0.001 |

^a^Model 1: unadjusted;

^b^Model 2: adjusted for age, sex, SBP, DBP;

^c^Model 3: adjusted for age, sex, SBP, DBP, CRP, TC, HDL-C, LDL-C, smoking, drinking, hypertension, hyperlipidemia, use of antihypertensives, and use of antilipidemic.

**Table S2. Correlation between the TyG index and the echogenicity of carotid artery plaque**

| Carotid plaque echogenicity | Variables | OR (95 %CI)^a^ | *P-*value | OR (95 %CI)^b^ | *P-*value | OR (95 %CI)^c^ | *P-*value |
| --- | --- | --- | --- | --- | --- | --- | --- |
| Hypoechoic | TyG index | 1.18 (1.12-1.24) | < 0.001 | 1.25 (1.18-1.32) | < 0.001 | 1.29 (1.20-1.39) | < 0.001 |
|  | Q1 | 0.80 (0.74-0.88) | < 0.001 | 0.74 (0.68-0.81) | < 0.001 | 0.84 (0.76-0.93) | 0.001 |
|  | Q2 | 0.99 (0.91-1.08) | 0.852 | 0.99 (0.91-1.08) | 0.825 | 0.99 (0.90-1.08) | 0.814 |
|  | Q3 | 1.14 (1.05-1.24) | 0.003 | 1.14 (1.05-1.24) | 0.003 | 1.06 (0.97-1.16) | 0.196 |
|  | Q4 | 1.14 (1.09-1.20) | < 0.001 | 1.18 (1.08-1.29) | < 0.001 | 1.14 (1.03-1.27) | 0.013 |
| Isoechoic | TyG index | 0.85 (0.79-0.92) | < 0.001 | 1.20 (1.15-1.26) | < 0.001 | 1.18 (1.10-1.26) | < 0.001 |
|  | Q1 | 0.93 (0.86-1.00) | 0.061 | 0.79 (0.73-0.85) | < 0.001 | 0.91 (0.84-1.00) | 0.044 |
|  | Q2 | 1.08 (1.00-1.17) | 0.045 | 0.93 (0.86-1.01) | 0.085 | 0.94 (0.87-1.02) | 0.144 |
|  | Q3 | 1.10 (1.01-1.20) | 0.027 | 1.08 (1.00-1.17) | 0.043 | 1.00 (0.92-1.09) | 0.966 |
|  | Q4 | 1.18 (1.09-1.27) | < 0.001 | 1.26 (1.16-1.36) | < 0.001 | 1.20 (1.10-1.32) | < 0.001 |
| Hyperechoic | TyG index | 1.12 (1.09-1.16) | < 0.001 | 1.28 (1.24-1.32) | < 0.001 | 1.33 (1.27-1.38) | < 0.001 |
|  | Q1 | 0.95 (0.90-0.99) | 0.013 | 0.81 (0.77-0.85) | < 0.001 | 0.92 (0.87-0.97) | 0.002 |
|  | Q2 | 0.89 (0.85-0.93) | < 0.001 | 0.88 (0.83-0.92) | < 0.001 | 0.89 (0.85-0.94) | < 0.001 |
|  | Q3 | 1.03 (0.99-1.08) | 0.171 | 1.04 (0.99-1.09) | 0.152 | 0.97 (0.92-1.02) | 0.224 |
|  | Q4 | 1.16 (1.11-1.21) | < 0.001 | 1.36 (1.30-1.43) | < 0.001 | 1.34 (1.26-1.42) | < 0.001 |
| Mixture | TyG index | 1.19 (1.15-1.23) | < 0.001 | 1.37 (1.32-1.42) | < 0.001 | 1.28 (1.22-1.34) | < 0.001 |
|  | Q1 | 0.83 (0.79-0.88) | < 0.001 | 0.70 (0.66-0.74) | < 0.001 | 0.86 (0.81-0.92) | < 0.001 |
|  | Q2 | 0.95 (0.90-1.00) | 0.058 | 0.94 (0.89-0.99) | 0.023 | 0.99 (0.94-1.05) | 0.788 |
|  | Q3 | 1.02 (0.97-1.08) | 0.476 | 1.04 (0.98-1.10) | 0.203 | 0.95 (0.90-1.00) | 0.086 |
|  | Q4 | 1.24 (1.18-1.31) | < 0.001 | 1.46 (1.38-1.55) | < 0.001 | 1.28 (1.20-1.37) | < 0.001 |

^a^Model 1: unadjusted;

^b^Model 2: adjusted for age, sex, SBP, DBP;

^c^Model 3: adjusted for age, sex, SBP, DBP, CRP, TC, HDL-C, LDL-C, smoking, drinking, hypertension, hyperlipidemia, use of antihypertensives, and use of antilipidemic.
